# Structure of Co-2 × 2 nanoislands grown on Ag/Ge(111)-√3 × √3 surface studied by scanning tunneling microscopy

**DOI:** 10.1186/1556-276X-7-189

**Published:** 2012-03-19

**Authors:** Xiao-Lan Huang, Chun-Liang Lin, Agnieszka Tomaszewska, Chun-Rong Chen, Tsu-Yi Fu

**Affiliations:** 1 Department of Physics, National Taiwan Normal University, 88, Sec. 4 Ting-Chou Rd., Taipei, 116, Taiwan, Republic of China

## Abstract

We have found that Co-2 × 2 islands grown on an Ag/Ge(111)-√3 × √3 surface have hcp structure with the (11-20) orientation. The island evolution involves transformation of the unit cell shape from parallelogram into rectangular, which is accompanied by the island shape transformation from hexagonal into stripe-like. Identified are two crystallographic directions for the island growth, the pseudo-[0001] and the pseudo-[1-100]. We have observed the occurrence of a lateral shift between the topmost and the underlying bilayers in the case of the island growth along the pseudo-[0001] direction. In contrast, the topmost and the underlying bilayers are unshifted for the growth along the pseudo-[1-100] direction.

## Introduction

Since its invention, scanning tunneling microscopy [STM] has served as a powerful means in providing an atomically resolved insight into the structure of solid surfaces [1]. An interest in surface structure exists in a strong correlation between the morphology of a particular surface and its electronic, optical, and magnetic properties [2]. Therefore, the need to characterize the surface morphology at the atomic level emerges in response to an increasing demand for materials with electronic and magnetic properties suitable for modern applications. Nowadays, in the face of tremendous progress in device miniaturization, the possibility to manipulate the properties of the surface seems to be particularly attracting. In principle, it may be achieved by depositing foreign atoms onto the surface, as it is known that numerous properties of the resultant epitaxial layers, such as lattice constant or electric conductance, are quite different from those of the substrate [3-6].

Among a palette of materials, the system Co/Ge represents a particularly promising case on account of a combination of the high-mobility substrate with a metal of an exceptionally high magnetization. However, a key concern is with Co-Ge intermixing, which results in the formation of non-ferromagnetic surface compounds.

An elegant solution which consists in Ag termination of the Ge(111) substrate surface and leads to the formation of a √3 × √3 reconstruction was proposed by Tsay et al. [7] for the growth of Co thin film. They have demonstrated that, in contrast to the non-ferromagnetic Co/Ge(111) surface, Co films grown on the Ag/Ge(111)-√3 × √3 substrate surface reveal magnetic properties and suggest that the intermediate Ag layer has buffering properties toward Co thin film growth by preventing the deposited Co atoms from a chemical reaction with the germanium surface.

The buffering properties of the Ag/Ge(111)-√3 × √3 surface may be accounted for in terms of its unique atomic arrangement, which is currently well established based on the experimental [8-14] and theoretical work [13]. The approved structural models commonly propose that the √3 × √3 surface has a structure in which both the Ag atoms and the outermost Ge atoms are arranged in a triangular configuration. The formation of a Ge triangle saturates two of three surface dangling bonds, and the remaining bond is saturated with an Ag atom. Owing to such an arrangement, the Co atoms cannot combine with Ge(111) surface atoms very easily, and hence, the surface remains inert toward the deposits.

The unique properties of the Co/Ag/Ge(111)-√3 × √3 surface inspired the work in our laboratory where, in the last several years, the attention has been focused on the STM characterization of nanosized Co structures (nanoclusters and nanoislands) grown on the surface under discussion [15-17]. We have found that, depending on coverage and annealing temperature, the Co islands reveal either √13 × √13 or 2 × 2 reconstruction. Dual-polarity STM images of individual structures indicate that the islands differ in conducting properties. That is, the structures with the √13 × √13 periodicity, having the empty-state image significantly different from the filled-state image, exhibit behavior typical for semiconductors. In contrast, islands with the 2 × 2 reconstruction reveal a metallic character with the empty-state image almost identical to the filled-state ones. This observation has led us to conclude that the reported magnetic properties of the Co/Ag/Ge(111)-√3 × √3 films should be ascribed to the Co-2 × 2 phase rather than that of the Co-√13 × √13.

The application importance of the magnetic Co-2 × 2 phase provides a practical motivation to conduct more profound studies on the island growth on the Ag/Ge(111)-√3 × √3 surface. For example, a complete picture of the Co-2 × 2 island growth has been hampered by a lack of a model for the island surface structure. However, the situation for Co epitaxy is rather complicated as Co can grow in three different structures, face-centered cubic [fcc], hexagonal close-packed [hcp], and body-centered cubic [bcc] [18].

In this work, based on our experimental observations, we propose that the Co-2 × 2 islands grow on the Ag/Ge(111)-√3 × √3 surface in an hcp structure and have a (11-20) crystallographic orientation. We hope that our findings may be useful for controlling the magnetic nanoisland growth on the surface.

## Experiment and methods

The Co-2 × 2 nanoislands were fabricated *in situ *with the use of an Omicron VT-STM (Omicron Taiwan R. O. C. Office Omega Scientific Taiwan Limited, Taipei, Taiwan, Republic of China) (base pressure approximately 2 × 10^-10 ^mbar) operating in constant-current mode and equipped with well-collimated evaporators for Ag and Co deposition. The Ge(111)-c2 × 8 surface was achieved by cleaning p-type Ge(111) wafers (1 to 10 Ω cm resistivity, 500 μm thickness) by repeated cycles of Ar^+ ^bombardment (1.0 keV, 10° to 90° incidence angle) followed by annealing at 920 K. The Ag/Ge(111)-√3 × √3 surface was prepared by exposing the substrate, kept at room temperature [RT], to an Ag beam from a K-cell dispenser for 90 min followed by annealing at 720 K. Then, the final surface was produced by Co deposition from an *e*-bombardment type evaporator for 30 min to obtain the coverage higher than 3 ML, which is suitable for fabrication of the desirable Co-2 × 2 phase. After deposition, the substrate was post-annealed at 670 K. All STM images presented in this paper were acquired at RT using KOH-etched W tips. The substrate temperatures were measured with a K-type thermocouple.

## Results and discussion

**Figure 1:**
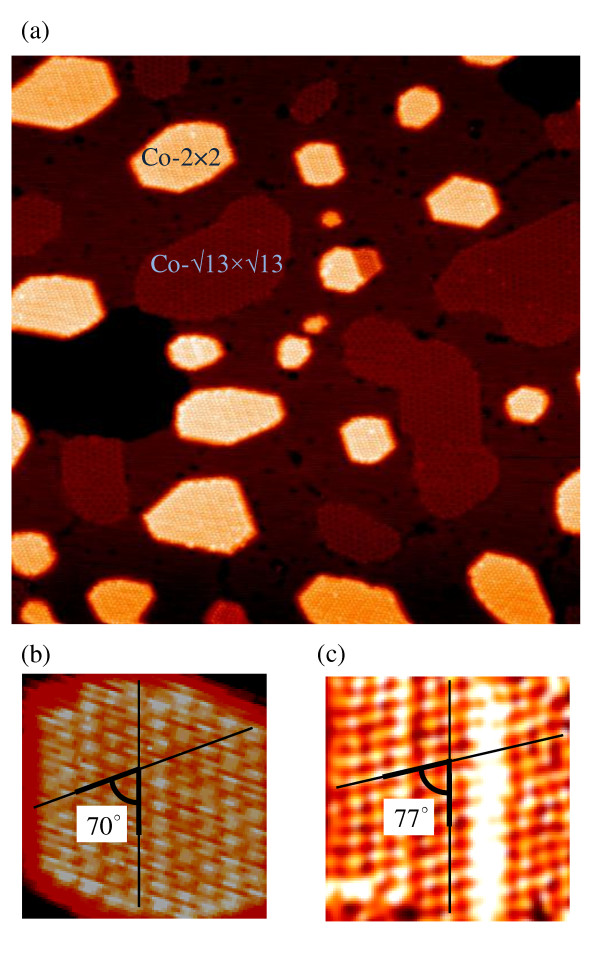
**STM images of Co islands on Ag/Ge(111)-√3 × √3 surface**. (**a**) 80 × 80-nm^2 ^STM image showing the coexistence of Co-2 × 2 and Co-√13 × √13 islands grown on Ag/Ge(111)-√3 × √3 surface. (**b**) 10 × 10-nm^2 ^STM image of hexagonal-shaped Co(11-20)-2 × 2 island of 0.4 nm height. (**c**) 10 × 10-nm^2 ^STM image of stripe-like Co(11-20)-2 × 2 island of 3.7 nm height. In b and c, the angles between the atomic rows are given.

Figure 1a shows a typical large-scale STM image of the surface which was prepared under the conditions described in the previous section. Most Co islands grown on the Ag/Ge(111)-√3 × √3 surface reveal the desirable 2 × 2 reconstruction at their tops. The contribution of the islands with the unwanted √13 × √13 reconstruction is only minor.

We found previously that the structural properties of the Co islands grown on the Ag/Ge(111)-√3 × √3 surface are strongly influenced by the structure of the Ge(111) surface [15]. This fact is caused by a very strong coupling between the Co atoms and the Ge(111) surface despite the presence of the Ag buffer layer. Therefore, in our further considerations, we neglect the presence of the Ag layer and propose a model for the Co-2 × 2 phase growth on the Ge(111) surface.

As seen in Figure 1a, the Co-2 × 2 islands adopt either hexagonal or stripe-like shapes. An insight into the inner structure of the individual islands is provided in Figure 1b, c. With respect to the shape, the unit cells of the hexagonal island (Figure 1b) at first glance seem to resemble parallelogram-shaped unit cells of the Ge(111)-1 × 1 surface, which indicates a strong influence of the substrate surface on the structure of the growing island. A closer inspection, however, reveals that the angle of the intersection between the island rows running in the directions shown in Figure 1b is larger as compared to that typical for the Ge(111)-1 × 1 surface (i.e., 70° vs. 60°). By analyzing a number of images, we have found that for the stripe-like islands, which are generally higher than their hexagonal counterparts, the above-mentioned discrepancy is even more distinct. For example, for the island shown in Figure 1c, the angle amounts to 77°. These observations have important implications for the model for the island growth. Namely, we can speculate that the island growth involves a distortion of the island rows with reference to the substrate surface rows. What is more, a degree of the distortion increases with the island height. As a consequence, the island unit cells undergo a shape transformation from initial parallelogram into rectangular. The alterations in the island inner structure are accompanied by noticeable changes in the island shape. That is, when the islands grow in height, the evolution proceeds to transform them from hexagonal-shaped into stripe-like-shaped. In view of that, in an attempt to construct the model for the Co-2 × 2 island growth, we focus on the stripe-like islands as a more evolved phase.

In order to ascribe a specific crystallographic structure to the Co-2 × 2 islands, we noted that the equilibrium phase for bulk Co is hcp at RT, and the Co layers generally adopt this structure when grown on metallic surfaces [19,20]. However, it was demonstrated that, under special preparation conditions, the Co layer can grow epitaxially on the Au(111) surface with the fcc structure [21-23]. Tonner et al. added more confusion to the issue by demonstrating that the occurrence of a particular structure depends on the thickness of deposited films. That is, while the initial Co growth on the clean Cu(111) proceeds in the fcc phase, the transition from fcc into hcp takes place as the film thickness increases beyond two layers [24].

Table 1 collates the values of the lattice parameters and the interlayer spacing for several low-index planes with bcc, fcc, and hcp structures of Co along with the corresponding values for the Ge(111)-1 × 1 surface. A comparison between the lattice parameters of the substrate with those of various Co surface phases leads us to identify the Co-hcp(11-20) and the Co-bcc(111) planes as those with the smallest lattice misfit to the substrate. In order to identify the actual phase, we have compared the unit cell shapes collected in Table 1 with those displayed in the STM images (Figure 1). The comparison leads us to conclude that the islands are oriented by the hcp(11-20) plane rather than the bcc(111) plane because the rectangular-shaped unit cells of the former resemble those typical for the evolved Co-2 × 2 islands.

**Table 1:** Geometrical characteristics of low-index Co crystallographic planes in bcc, fcc, and hcp structures.

Crystallographic orientation	Lattice pattern	Lattice parameters (pm)	Interlayer distance (pm)
Co-fcc(100)		251 × 251	177
Co-fcc(110)		251 × 355	125
Co-fcc(111)		251 × 251	205
Co-hcp(0001)		251 × 251	204
Co-hcp(11-20)		434 × 407	125
Co-hcp(10-10)		251 × 407	72 145
Co-bcc(100)		284 × 284	142
Co-bcc(110)		284 × 402	201
Co-bcc(111)		402 × 402	82 164
Ge(111)		400 × 400	82 245

bcc, body-centered cubic; fcc, face-centered cubic; hcp, hexagonal close-packed; pm, picometer.

**Figure 2:**
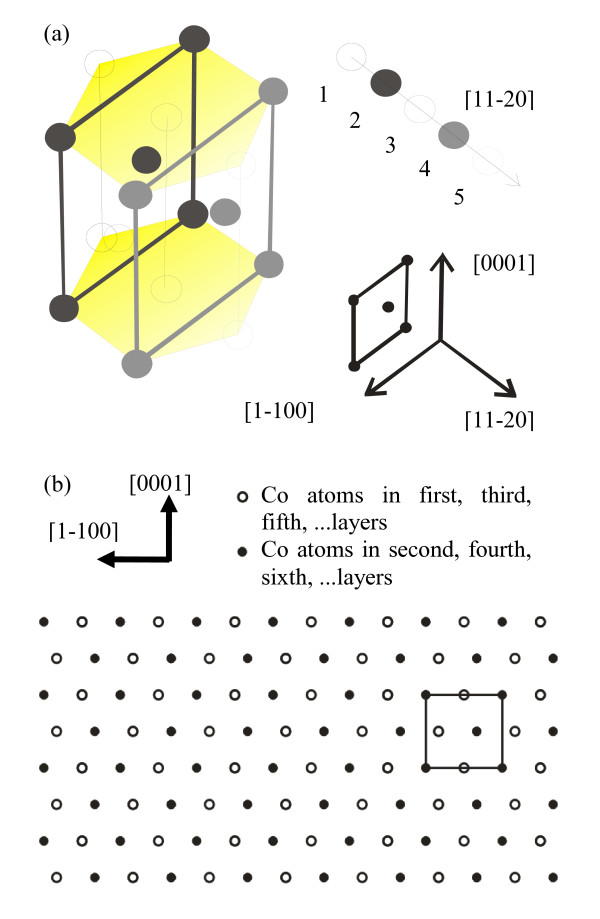
**Schematic diagram of hcp structure**. (**a**) Numbered balls represent atoms which belong to successive atomic layers, perpendicular to [11-20] crystallographic direction. The open circles refer to the layers with odd numbers, while the filled circles represent those with even numbers. Edges of hexagonal prism which belong to [0001] and [1-100] directions in the second layer are denoted with black line, while those in the fourth layer are shown by gray lines. (**b**) Array showing an atomic arrangement in the successive layers of the hcp structure (a top view). The open circles refer to the atoms in the odd layers, while those in the even layers are represented by the filled circles. Two crystallographic directions are denoted.

Figure 2a provides a schematic diagram of the hcp structure. The lattice parameters for the Co(11-20)-1 × 1 unit are 407 pm along the [0001] direction and 434 pm along the [1-100] direction. The value for the interlayer spacing is by far lower than the lattice parameters (i.e., 125 pm), indicating that the islands are more likely to form bilayered structures than single-layered ones. As it is schematically shown in Figure 2b, for the (11-20) surface, the atomic positions in the first, third, and fifth layers are identical as viewed from the top. Similarly, the atomic configuration in the second layer is repeated in the fourth and sixth layers. For further considerations, we assume that the Co islands are composed of bilayers, which means that the atomic positions in each layer are equivalent.

**Figure 3:**
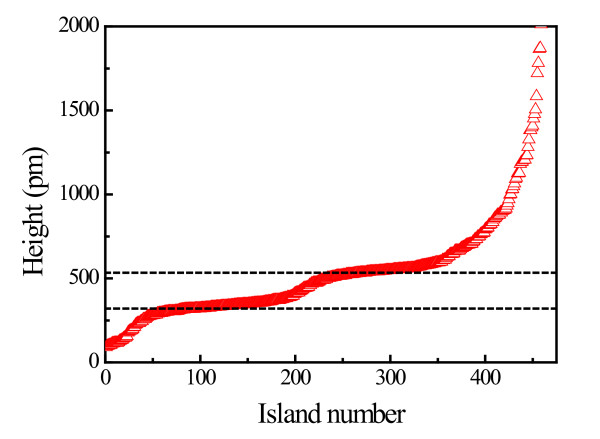
**Distribution of height within a population of 500 Co-2 × 2 islands**.

In order to verify our assumption, we have measured line profiles along the Co-2 × 2 islands randomly chosen from a number of large-scale STM images acquired under similar tunneling conditions. Then, the islands have been numbered in increasing order from the lowest to the highest. If two or more islands have identical height, we have ascribed them not the same, but successive numbers. The obtained data are presented in Figure 3. It is clearly seen that the majority of islands have the height equal to either 320 or 535 pm. However, it seems questionable to assume that the figures represent real island heights. First, in spite of the fact that the intermediate Ag layer does not influence the structural properties of the Co islands, the presence of the layer cannot be neglected in the interpretation of the height measurements. In order to minimize the errors caused by the presence of the Ag layer of unknown thickness, we propose to use a difference between the measured values as a piece of evidence for the formation of the bilayered Co-2 × 2 structures. The discrepancies between the measured height difference (i.e., 215 pm) and the theoretical height of the Co-(11-20) bilayer (i.e., 250 pm) must be due to the tip-caused effects which frequently appear when the imaging objects differ in conducting properties. In the case of a bimetal/semiconductor system, the above-mentioned issue is even more critical.

**Figure 4:**
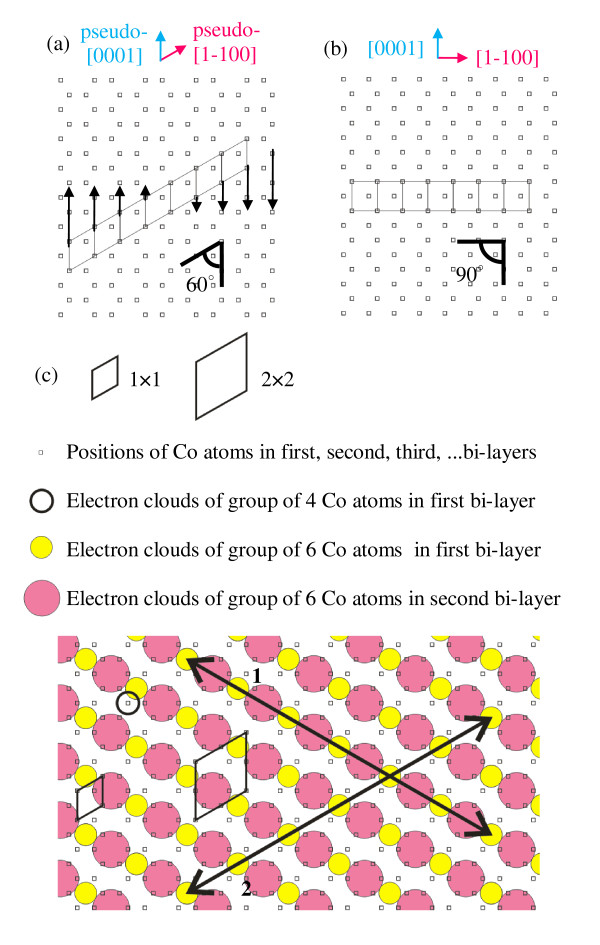
**Schematic diagrams of Co-(11-20) crystallographic planes**. (**a**) Array of parallelogram-shaped Co-1 × 1 unit cells. Direction of row shift is marked with arrows. (**b**) Array of rectangular Co-1 × 1 unit cells. (**c**) Array of parallelogram Co-2 × 2 unit cells against a background of Co-1 × 1 lattice. The filled circles refer to electron clouds of groups of six neighboring atoms, while the open circle corresponds to a group of four neighboring atoms. Two different directions for the island growth are marked with numbered arrows.

As we proposed above, the Ge(111)-1 × 1 surface acts as a template for the Co island growth. We can imagine the template as an array of parallelogram-shaped unit cells, similar to that shown in Figure 4a. In the early stages, the unit cells of the growing Co island adopt the shape similar to that typical for the unit cells of the substrate surface. Then, the island evolution is realized by the distortion of the island rows with reference to the substrate surface rows (in Figure 4a, the direction of the distortion is indicated by arrows). As a result, the island unit cells adopt the rectangular shape. A schematic of the resultant Co-1 × 1 structure is shown in Figure 4b. The weakness of the proposed model exists, however, in the lack of the correspondence between the predicted Co island periodicity and that observed in the STM images (i.e., Co-1 × 1 vs. Co-2 × 2). In an attempt to account for this issue, we shall recall some recent findings which have shown that, in the case of metal/semiconductor epitaxial system, the protrusions which appear in the STM image do not represent individual atoms but correspond to the sites at which electron clouds of groups of atoms concentrate [25]. In order to identify the sites which appear as protrusions in our STM images, we consider two alternative positions. In Figure 4c, the larger filled circles represent the sites at which electron clouds of groups of six neighboring atoms are likely to accumulate. The small open circle refers to the alternative site at which an electron cloud of a group of four neighboring atoms is accumulated. It is obvious that, due to a smaller number of constituting atoms, the latter groups have a lower probability to be detected by the tip, as compared to former. If the sites indicated by the small open circle are not visible in the STM image, then the protrusions form a distinct 2 × 2 pattern, which must be due to the sites enclosed with larger filled circles.

**Figure 5:**
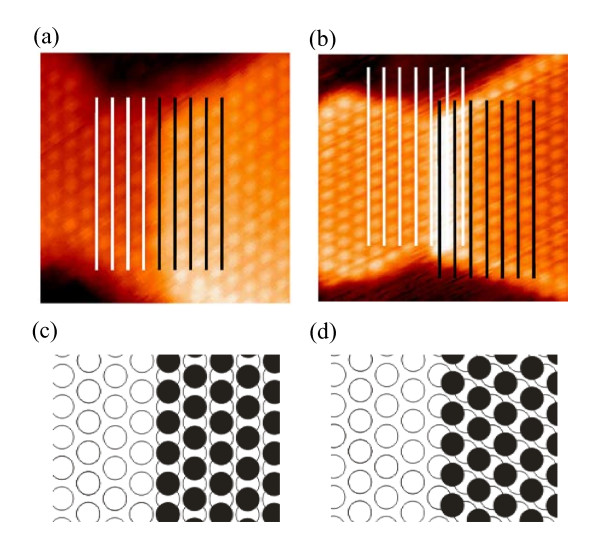
**STM images and the corresponding diagrams of Co islands grown along pseudo-[0001] and pseudo-[1-100] directions**. (**a**)10 × 10-nm^2 ^STM images showing Co-(11-20)-2 × 2 islands growing along the pseudo-[1-100] direction, and (**b**) the pseudo-[0001] direction. The black lines represent the rows in the topmost bilayers while the white lines refer to the rows in the underlying layers. Corresponding schematic diagrams are shown in (**c**) and (**d**), respectively. The filled circles refer to the protrusions in the topmost bilayer, while the open circles represent the atomic protrusions in the underlying bilayers.

We shall underline that, although the atomic positions in the successive (11-20) bilayers are identical, this is not the case for the sites at which electron clouds are accumulated. In Figure 4c, the smaller filled circles represent the sites at which electron clouds of groups of six neighboring Co atoms positioned in the lower bilayer are accumulated. Due to charge repulsion, electron clouds of similar groups of atoms from the topmost bilayer are shifted to the sites enclosed with the larger filled circles. In Figure 4c, we can easily notice the existence of two inequivalent directions for the island growth (denoted as 1 and 2). If the island grows along the direction 1, then the rows in the topmost bilayer are unshifted with respect to the rows in the underlying bilayer. In contrast, if the island growth proceeds through the direction 2, then the topmost and the underlying bilayers are shifted with respect to each other. Figure 5 summarizes atomically resolved STM images of the Co(11-20)-2 × 2 islands grown along the direction 1 (Figure 5a) and the direction 2 (Figure 5b) along with illustrative schematic diagrams (Figure 5c, d). In Figure 5a, b, black lines refer to the rows in the topmost bilayers, while the rows in the underlying bilayers are indicated by white lines. In Figure 5c, d, filled and open circles refer to the protrusions in the topmost and the underlying bilayers, respectively. We can clearly observe the lateral shift between the bilayers when the island grew along the direction 2, but the shift is nonexistent for the island growth along the direction 1. From statistical analysis, we have found that 90% of the Co(11-20)-2 × 2 islands were grown along the direction 2, suggesting that for this case the lattice misfit between the island and the substrate is the smallest. We can notice that the lattice misfit between the rectangular-shaped Co(11-20)-2 × 2 and the Ge(111)-1 × 1 unit cell amounts to 2% along the [0001] direction but 9% along the [1-100] one. Therefore, we ascribe the direction 2 to the [0001] direction and the direction 1 to the [1-100] direction. Since the unit cells in the observed islands do not have ideal rectangular shapes, we ascribe the directions to the pseudo-[0001] direction and the pseudo-[1-100] direction.

**Figure 6:**
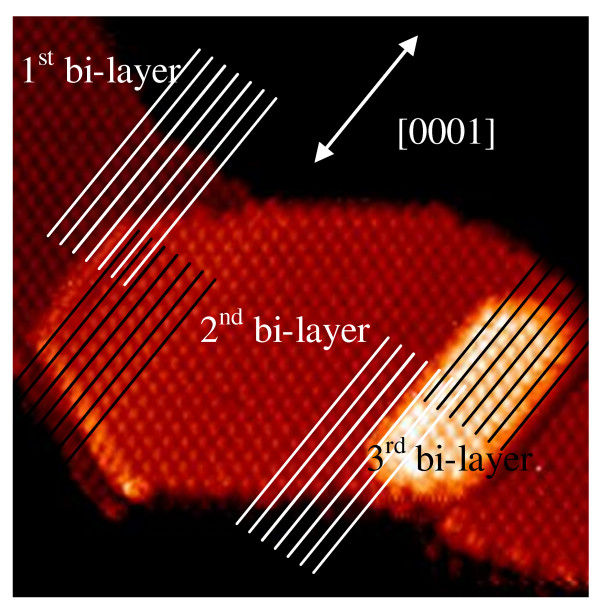
**STM image showing the Co-(11-20)-2 × 2 island which is composed of three bilayers**. Lateral shifts between the successive bilayers indicate that the island grew along the pseudo-[0001] direction.

So far, we have focused on the Co-2 × 2 islands composed of two bilayers. However, some Co-2 × 2 islands observed in the STM images constitute multi-bilayered systems. Figure 6 provides an image of the island which is composed of three bilayers. More detailed information about the growth of this system may be extracted from the examination of the structure which appears at the boundary between the successive bilayers. In particular, it is of interest to find out whether for such a system each bilayer grows along the same direction. An inspection at the boundary between the first and the second bilayers reveals the appearance of a lateral shift, indicating that the bilayers grew along the pseudo-[0001] direction. Similarly, the boundary between the second and the third bilayers has been scrutinized, and the presence of a similar lateral shift has been found. This observation indicates that, for a particular island, there exists only one preferential direction for the growth. By analyzing a number of similar multi-bilayered islands, we have found that this is indeed true for the Co(11-20)-2 × 2 islands grown on the Ag/Ge(111)-√3 × √3 surface.

## Conclusions

We have observed that the Co-2 × 2 islands grown on the Ag/Ge(111)-√3 × √3 surface reveal hexagonal and stripe-like shapes. They commonly grow in the hcp structure and are oriented by the (11-20) face. However, in view of the island growth, the hexagonal islands may be regarded as a less evolved stage from which the stripe-like islands develop. The island shape evolution is accompanied by a transition of the unit cell shape from parallelogram-like into rectangular. In the majority of cases, the islands grow along the pseudo-[0001] crystallographic direction, giving the lattice mismatch of 2% between the growing phase and the substrate. Some 10% of islands grow along the pseudo-[1-100] direction for which the lattice mismatch amounts to 9%.

## Abbreviations

bcc: body-centered cubic; fcc: face-centered cubic; hcp: hexagonal close-packed; pm: picometer; RT: room temperature; STM: scanning tunneling microscopy.

## Competing interests

The authors declare that they have no competing interests.

## Authors' contributions

XLH conceived of the study, analyzed the data, elaborated the model, and wrote the manuscript. CLL was involved in the conception of the study. AT was involved in the drafting of the manuscript and the conception of the study. CRC carried out the experiment. TYF is the principal investigator. All authors read and approved the final version of the manuscript.
